# Nationwide genetic analysis of more than 600 families with inherited eye diseases in Argentina

**DOI:** 10.1038/s41525-023-00352-1

**Published:** 2023-05-22

**Authors:** Patricio G. Schlottmann, José D. Luna, Natalia Labat, María Belén Yadarola, Silvina Bainttein, Evangelina Esposito, Agustina Ibañez, Evangelina Ivón Barbaro, Alejandro Álvarez Mendiara, Carolina P. Picotti, Andrea Chirino Misisian, Luciana Andreussi, Julieta Gras, Luciana Capalbo, Mauro Visotto, José E. Dipierri, Emilio Alcoba, Laura Fernández Gabrielli, Silvia Ávila, María Emilia Aucar, Daniel M. Martin, Gerardo Juan Ormaechea, M. Eugenia Inga, Aníbal A. Francone, Martin Charles, Tamara Zompa, Pablo Javier Pérez, Vanesa Lotersztein, Pedro J. Nuova, Ivana B. Canonero, Omar A. Mahroo, Michel Michaelides, Gavin Arno, Malena Daich Varela

**Affiliations:** 1 Charles Centro Oftalmológico, Buenos Aires, Argentina; 2 Centro Privado de Ojos Romagosa SA, Córdoba, Argentina; 3 Instituto Oftalmológico de Córdoba, Córdoba, Argentina; 4 University Clinic Reina Fabiola, Córdoba, Córdoba, Argentina; 5 Catholic University of Cordoba, Cordoba, Argentina; 6 Hospital Provincial Neuquén, Neuquén, Argentina; 7 Instituto Oftalmológico Cortina, Santa Rosa, La Pampa Argentina; 8 Centro Médico Lisandro de la Torre, Villa María, Córdoba, Argentina; 9 Clínica de la Visión, San Juan, Argentina; 10 Centrovision Mendoza SA, Mendoza, Argentina; 11 Hospital MJ Becker, La Punta, San Luis, Argentina; 12 Instituto Oftalmológico Trelew, Trelew, Chubut Argentina; 13 Universidad Nacional de Jujuy, Jujuy, Argentina; 14 Hospital Materno Infantil Dr Héctor Quintana, Jujuy, Argentina; 15 Nuevo Hospital San Antonio de Padua, Río Cuarto, Córdoba, Argentina; 16 Facultad de Ciencias Médicas, Universidad Nacional del Comahue, Río Negro, Argentina; 17 Instituto de Ojos y Oídos, Resistencia, Chaco Argentina; 18 Clínica de Ojos Córdoba, Córdoba, Argentina; 19 Organización Medica de Investigación, Buenos Aires, Argentina; 20 Santiago del Estero, Argentina; 21 Centro Nacional de Genética Médica, Buenos Aires, Argentina; 22 Ocularyb Oftalmoclinica, Yerba Buena, Tucumán, Argentina; 23 Hospital Privado Universitario de Córdoba, Córdoba, Argentina; 24 Moorfields Eye Hospital, London, UK; 25 UCL Institute of Ophthalmology, University College London, London, UK

**Keywords:** Genetic testing, Genetic testing

## Abstract

This study corresponds to the first large-scale genetic analysis of inherited eye diseases (IED) in Argentina and describes the comprehensive genetic profile of a large cohort of patients. Medical records of 22 ophthalmology and genetics services throughout 13 Argentinian provinces were analyzed retrospectively. Patients with a clinical diagnosis of an ophthalmic genetic disease and a history of genetic testing were included. Medical, ophthalmological and family history was collected. A total of 773 patients from 637 families were included, with 98% having inherited retinal disease. The most common phenotype was retinitis pigmentosa (RP, 62%). Causative variants were detected in 379 (59%) patients. *USH2A*, *RPGR*, and *ABCA4* were the most common disease-associated genes. *USH2A* was the most frequent gene associated with RP, *RDH12* early-onset severe retinal dystrophy, *ABCA4* Stargardt disease, *PROM1* cone-rod dystrophy, and *BEST1* macular dystrophy. The most frequent variants were *RPGR* c.1345 C > T, p.(Arg449*) and *USH2A* c.15089 C > A, p.(Ser5030*). The study revealed 156/448 (35%) previously unreported pathogenic/likely pathogenic variants and 8 possible founder mutations. We present the genetic landscape of IED in Argentina and the largest cohort in South America. This data will serve as a reference for future genetic studies, aid diagnosis, inform counseling, and assist in addressing the largely unmet need for clinical trials to be conducted in the region.

## Introduction

**Fig. 1:**
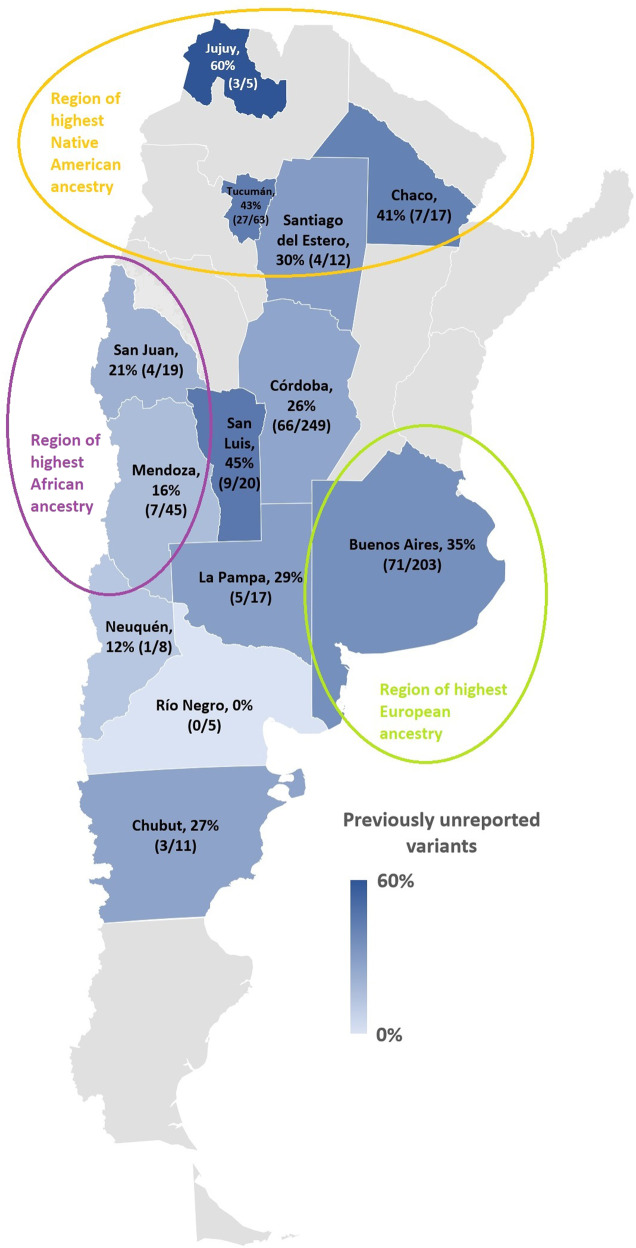
Map of Argentina, provinces in shades of blue participated in the present study. The blue gradient represents the percentage of previously unreported/total variants in each province, with darker tones corresponding to higher percentage and lighter, lower. Of note, the province with the highest percentage (60%, Jujuy) contained only a few variants and cases, possibly representing a bias.

The Latino population has diverse genetic ancestry that includes Native American, Asian, European, West African, and other minorities such as Jewish^1^. Argentina has received multiple migratory currents from Europe (mostly Spain and Italy), who also brought enslaved peoples from West Africa. The Argentinian population is reported to have 67% European, 28% Native American, 4% West African, and 1% East Asian ancestry^2^. Given it is the second largest country in South America, the genetic heterogeneity between regions is statistically significant, with European ancestry being the largest in Buenos Aires (76%) and the lowest in the North–West (33%)^3,4^. African roots are highest in the center of the country (Mendoza, San Juan), and Native American ancestry prevails in the North–West & Chaco (Fig. 1).

Genetics is one of the fastest-growing fields in healthcare, with substantial technological advancements during the last decades^5^. Initially, access to genetic testing further expanded the disparity between those with access to quality healthcare and those without^6^. As the cost of testing has decreased, worldwide access has improved, including being covered by the public national healthcare systems in countries such as the United Kingdom.

The sequencing of the first human genome was used to create a standard reference (currently GRCh38), based on 11 individuals from African and white backgrounds^7,8^. The previous version, GrCh37, is thought to have an ancestral make-up of 57% European, 37% African American and 6% East Asian^8,9^. Even though these constructs are mostly adequate for clinical and research purposes, the lack of diversity and the use of such reference for other ethnicities has been questioned^10^. Furthermore, the inequitable representation in genomic research leads to increased incidence of variants of uncertain significance (VUS) amongst individuals from ethnic minorities^11–13^. This disparity leads to difficulties in variant interpretation, genetic counseling, and the need of further exploration, all potentially more challenging in these often less affluent populations.

In the current era of thriving genetic therapies especially in the ophthalmic genetics field^14,15^, developing countries are starting to share data from their own regions, contributing with previously unreported disease-causing variants, atypical presentations, and detailed longitudinal information^16–19^.

In this study, we present the largest South American cohort of genetically confirmed families with inherited eye diseases (IED); an important and timely addition to the global IED genomic dataset.

## Results

### Demographics and clinical diagnosis

Seven hundred and seventy-three patients from 637 families were included in the study. Three hundred and eighty-six patients (50%) were female and 387 (50%) were male. Amongst those who declared ethnicity (96%), 371 (50%) were white, 299 (40%) were Hispanic or Latino, 66 (9%) were Native Americans, and 6 (1%) were mixed. Two hundred and fifty-eight patients (33%) declared a positive family history of similar eye disease.

The mean age of onset was reported to be 14.8 ± 13.1 years old (birth—82 years range), the mean age at diagnosis was 22.4 ± 15.7 years old (birth—82 years), and the mean age at genetic testing was 36.5 ± 18.9 (6 months old—83 years). The mean time between symptoms onset and diagnosis was 4.3 ± 9.6 years (0–81), between disease onset and genetic testing, 14.9 ± 17.1 (0–82), and between diagnosis and genetic testing, 11.5 ± 14.6 (0–75).

**Fig. 2:**
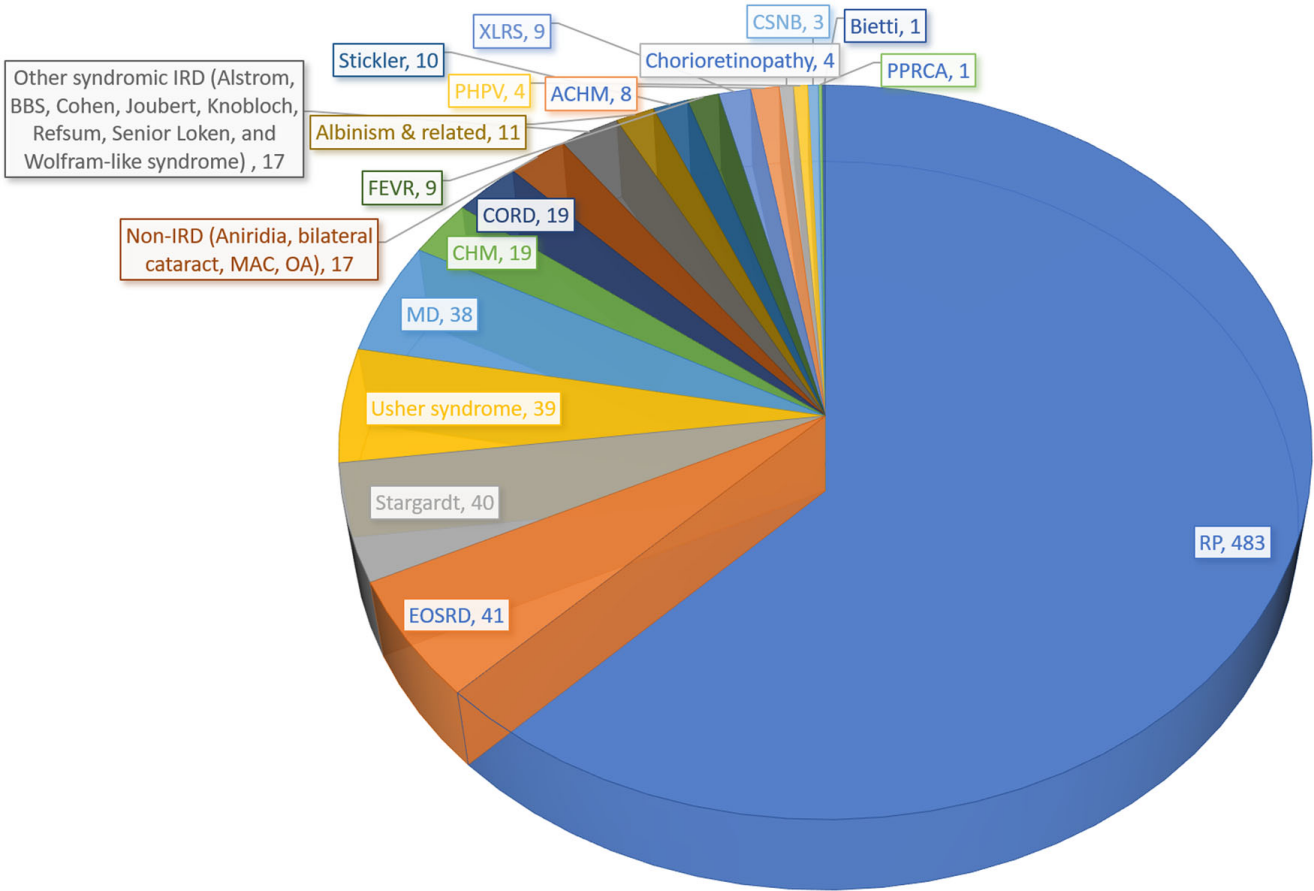
Clinical diagnoses of 773 patients with inherited eye diseases in Argentina. RP retinitis pigmentosa, EOSRD early-onset severe retinal dystrophy, MD macular dystrophy, CHM choroideremia, CORD cone-rod dystrophy, IRD inherited retinal dystrophy, BBS Bardet–Biedl syndrome, FEVR familial exudative vitreoretinopathy, XLRS X-linked retinoschisis, MAC microphthalmia-anophthalmia-coloboma spectrum, PHPV persistent hyperplastic primary vitreous, CSNB congenital stationary night blindness, PPRCA pigmented paravenous retinochoroidal atrophy, OA optic atrophy. Albinism & related conditions include oculo-cutaneous albinism, ocular albinism, and foveal hypoplasia.

Seven hundred and fifty-six patients (98%) had inherited retinal diseases (IRD), and the remaining 17 (2%) had other etiologies, such as optic atrophy (6) and coloboma (4). Four hundred and eighty-three patients (62%) had a diagnosis of non-syndromic retinitis pigmentosa (RP), 41 (5%) of early-onset severe retinal dystrophy (EOSRD), 40 (5%) of Stargardt disease, 39 (5%) of Usher syndrome, 38 (5%) of macular dystrophy (MD), 19 (2%) of cone-rod dystrophy (CORD), 19 (2%) of choroideremia (CHM), and the remaining patients had less frequent conditions (Fig. 2 and Supplementary Table 1).

### Genetics

#### Cohort

Of 637 families, (i) 379 (59%) had a definitive genetic testing result and were considered genetically solved (“positive”), (ii) 178 (28%) had negative testing, (iii) 42 (7%) had only one disease-causing variant in a recessive gene, and (iv) 38 (6%) harbored one or more VUS.

There was no significant difference between the age of onset, age at diagnosis, and age at genetic testing between these four groups (ANOVA *P=* 0.2621, 0.0654, and 0.6613, respectively). Positive family history was declared by 40% of individuals in the positive genetic testing group, 29% in the negative group, 23% in the one candidate variant, and 29% in the VUS.

**Fig. 3:**
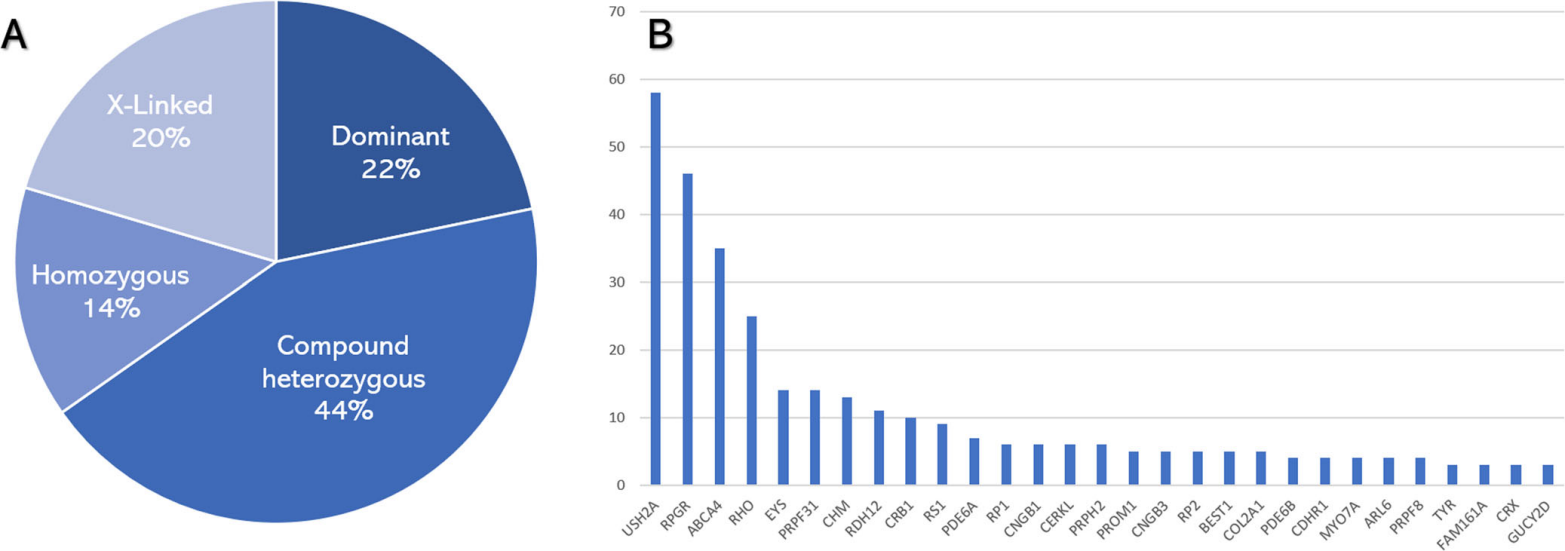
Inheritance patterns and most common genes in the cohort. **A** Pie graph representing the genotypes found in our cohort. **B** Bar graph showing the most frequently seen genes in the cohort, ranked by the number of affected families. The remaining genes were present in one or two families nationwide.

**Fig. 4:**
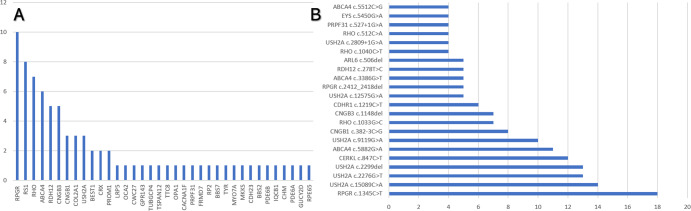
Most common genes in pediatric patients and most frequently found variants in the complete cohort. **A** Bar graph showing the most frequently found genes in patients under 18 years of age, ranked by the number of affected families. **B** Bar graph showing the most frequently found variants in the cohort, ranked by the number of alleles. The remaining variants were present in up to three families nationwide.

In the genetically solved group (*n* = 379), 220 had autosomal recessive inheritance (58%, 166 compound heterozygous and 54 homozygous), 82 (22%) had an autosomal dominant inheritance, and 77 (20%) were X-linked (Fig. 3A). The most common disease-causing genes were *USH2A* in 58 families, *RPGR* in 46, *ABCA4* in 35, *RHO* in 25, *PRPF31* and *EYS* in 14 each, *CHM* in 13, *RDH12* in 11, *CRB1* in 10, and the remaining cases appeared in less than 10 families nationwide (Fig. 3B). *USH2A* was the most common gene to cause RP, *RDH12* EOSRD, *ABCA4* Stargardt, *PROM1* CORD, and *BEST1* MD. In the pediatric cohort (under 18 years of age), the most common genes were *RPGR* (*n* = 10 families), *RS1* (*n* = 8), *RHO* (*n* = 7), *ABCA4* (*n* = 6), and *RDH12* and *CNGB3* (*n* = 5 each, Fig. 4A).

#### Sequence variants

Four hundred and forty-eight different sequence variants were detected in the entire cohort: 193 (43%) missense, 107 (24%) frameshift insertion and/or deletions, 70 (16%) nonsense, 30 (7%) splice site (−2 to +5), 25 (6%) copy number/structural variants, 11 (2%) deep intronic, 8 (2%) inframe insertions and/or deletions, and 4 (1%) synonymous changes.

The most common variants were *RPGR* c.1345 C > T, p.(Arg449*), present in 18 alleles of 18 unrelated families; *USH2A* c.15089 C > A, p.(Ser5030*) in 14 alleles of 14 families; *USH2A* c.2299del, p.(Glu767Serfs*21) and c.2276 G > T, p.(Cys759Phe) in 13 alleles of 13 families each; *CERKL* c.847 C > T, p(.Arg283*) in 12 alleles of 6 families; *ABCA4* c.5882 G > A, p.(Gly1961Glu) in 11 alleles of 11 families, and *USH2A* c.9119 G > A, p.(Trp3040*) in 10 alleles of 7 families (Fig. 4B).

The most common variant combinations in recessively inherited genes were *CERKL* homozygous c.847 C > T, p(.Arg283*) in 6 families; *USH2A* c.15089 C > A, p.(Ser5030*) and c.2299del, p.(Glu767Serfs*21) in 4 each; and *CDHR1* homozygous c.1219 C > T, p.(Arg407*), *CNGB3* homozygous c.1148del p.(Thr383Ilefs*13), and *USH2A* homozygous c.9119 G > A, p.(Trp3040*) in 3 families each.

One hundred and ninety-four previously unreported variants (43%) were identified in our cohort; with 156 classified as pathogenic/likely pathogenic and 38 as VUS (Table 1 and Supplementary Table 2). Twenty-one were found in *USH2A*, 18 in *RPGR*, 13 in *EYS*, 8 in *ABCA4*, 9 in *PRPF31*, and others in less frequent genes (Table 1). *RPGR* c.2412_2418del (p.Gly805Lysfs*8), *CDHR1* c.1219 C > T (p.Arg407*), *CNGB1* c.382-3 C > G, and *ARL6* c.506del (p.Gly169Alafs*6) were each present in 5 unrelated families; and *PROM1* c.1956T > G (p.Tyr652*), *EYS* c.6131_6134del (p.Asn2044Thrfs*11), *RPGR* c.2405_2406del (p.Glu802Glyfs*32), and *PRPF31* c.527 + 1 G > A were in three unrelated families, each; possibly representing founder mutations.

**Table 1:** Previously unreported disease-causing variants in our cohort.

Gene	Diagnosis	Variant c.	Variant p.	Gene	Diagnosis	Variant c.	Variant p.
***ABCA4***	Stargardt	c.302+4A>G	NA	***MKKS***	BBS	c.1013C>A	p.(Ser338*)
		c.614G>A	p.(Cys205Tyr)	***PCARE***	RP	c.1827del	p.(Gln610Argfs*135)
		c.1240-1G>A	NA	***PDE6A***	RP	c.1117G>T	p.(Glu373*)
		c.1919C>G	p.(Pro640Arg)			c.1538del	p.(Leu513Glnfs*7)
		c.3564_3566delinsAAG	p.(Cys1188*)			c.1955_1974dup	p.(Ile659Valfs*10)
		c.4877C>A	p.(Ala1626Asp)			c.2135+1G>T	NA
		Deletion Exons 39-40	NA			Deletion Exon 6	NA
		c.5835+1G>T	NA			Deletion Exon 9	NA
***ADGRV1***	USH2	c.11563G>T	p.(Glu3855*)	***PDE6C***	ACHM	c.295T>C	p.(Phe99Leu)
		c.13758_13761del	p.(Gly4587Glufs*2)			c.2036+1G>C	NA
***AGBL5***	RP	c.421dup	p.(His141Profs*23)	***PHYH***	Refsum syndrome	c.380A>G	p.(Asp127Gly)
***AHI1***	RP	c.3196C>T	p.(Arg1066*)	***PROM1***	RP	c.1956T>G	p.(Tyr652*)
***ALMS1***	Alstrom syndrome	c.542_545dup	p.(Asp182Glufs*4)			c.2490-2A>G	NA
		c.9784+1G>C	NA		CORD	Deletion Exon 16	NA
***ARL6***	RP	c.344A>G	p.(His115Arg)			c.2489+1G>A	NA
		c.350-2A>C	NA	***PRPF31***	MD	c.523C>T	p.(Gln175*)
		c.506del	p.(Gly169Alafs*6)			c.901_919del	p.(Leu301Valfs*14)
***ARSG***	USH4	c.1010G>A	p.(Trp337*)		RP	c.23T>G	p.(Leu8*)
***BBS4***	BBS	c.777_778del	p.(Tyr259*)			c.221_224dup	p.(Lys76fs)
***BBS7***	BBS	c.785_786del	p.(Asp262fs)			c.455del	p.(Asn152Metfs*46)
		c.947G>T	p.(Gly316Val)			c.527+1G>A	NA
***BEST1***	Vitelliform MD	c.13T>A	p.(Tyr5Asn)			c.749dup	p.(Met250Ilefs*29)
***CACNA1F***	CSNB	c.2269G>C	p.(Glu811Gln)			c.795del	p.(Ser266Glnfs*55)
***CDH23***	USH1	c.336del	p.(Asp109fs)			c.1263dup	p.(Lys422Glnfs*53)
		Deletion Exons 17-19	NA	***PRPH2***	MD	c.646C>T	p.(Pro216Ser)
		c.2801C>T	p.(Pro934Leu)	***REEP6***	EOSRD	c.481C>T	p.(Arg161*)
		c.7832_7833del	p.(Phe2611Cysfs*31)	***RHO***	RP	c.330C>G	p.(Cys110Trp)
***CDHR1***	RP	c.1219C>T	p.(Arg407*)			c.760_762dup	p.(Val254dup)
		c.1956del	p.(Trp652fs)			c.889A>C	p.(Ser297Arg)
***CEP290***	EOSRD	c.734_735del	p.(Glu245Valfs*10)	***RP1***	RP	c.532C>T	p.(Gln178Ter)
		c.4945C>T	p.(Gln1649*)			c.1299_1306dup	p.(Gln436Leufs*22)
***CHM***	CHM	c.546T>A	p.(Cys182*)			c.2555del	p.(Lys852Argfs*4)
		c.561T>A	p.(Cys187*)			c.3416del	p.Lys1139fs
		c.702+3_702+12del	NA			c.5564del	p.(Lys1855Argfs*42)
		c.1066A>T	p.(Lys356*)	***RP2***	RP	Deletion Exons 1-3	NA
		c.1674del	p.(Asp559Thrfs*24)			c.465_468dup	p.(Phe157Serfs*18)
***CNGB1***	RP	c.382-3C>G	NA	***RPE65***	EOSRD	c.314C>T	p.(Thr105Ile)
		c.1276del	p.(Glu426Argfs*77)	***RPGR***	CORD	c.3092_3093del	p.(Glu1031Glyfs*47)
		c.2030G>A	p.(Arg677His)			c.3218_3236dup	p.(Glu1075Valfs*10)
***COL18A1***	RP	c.1765G>T	p.(Gly589*)			c.3348del	p.(Glu1117Serfs*14)
***COL2A1***	Stickler	c.233dup	p.(Glu79*)		RP	c.356T>C	p.(Leu119Ser)
		c.1995+1G>T	NA			c.823G>T	p.(Gly275Cys)
***CRB1***	EOSRD	c.596C>A	p.(Ala199Asp)			c.1872_1873del	p.(Glu624Aspfs*5)
		c.3708_3709dup	p.(Ser1237Phefs*46)			c.2234_2237del	p.(Arg745Lysfs*69)
	RP	c.750T>A	p.(Cys250*)			c.2405_2406del	p.(Glu802Glyfs*32)
		c.1172-2A>G	NA			c.2412_2418del	p.(Gly805Lysfs*8)
		c.2053G>A	p.(Gly685Arg)			c.2442_2445del	p.(Gly817Lysfs*2)
		c.2784T>G	p.(Cys928Trp)			c.2501del	p.(Glu834Glyfs*255)
***CRX***	EOSRD	c.591_594dup	p.(Ser199fs)			c.2527del	p.(Glu843Lysfs*246)
***CWC27***	RP	c.495G>A	p.(Glu165=)			c.2543delA	p.(Glu848Glyfs*241)
		c.1101T>G	p.(Tyr367*)			c.2819_2837dup	p.(Glu947fs)
***EYS***	RP	c.514C>T	p.(Gln172*)			c.2964_2965del	p.(Glu989Glyfs*89)
		c.618_619del	p.(Ser207Trpfs*8)	***RPGRIP1***	RP	c.2910_2911del	p.(Pro971fs)
		c.2527G>A	p.(Gly843Arg)	***RS1***	XLRS	c.78+5G>C	NA
		Deletion Exons 17-22	NA			c.214G>A	p.Glu72Lys
		Deletion Exon 22	NA	***RTN41P1***	RP + OA	c.968_972dup	p.(Gly325Leufs*2)
		c.3938T>A	p.(Leu1313*)	***TSPAN12***	FEVR	c.916del	p.(Ter306fs)
		c.6131_6134del	p.(Asn2044Thrfs*11)	***TTC8***	BBS	c.991C>T	p.(Gln341*)
		c.6812_6813del	p.(Thr2271Argfs*11)	***TTLL5***	RP	c.1270C>T	p.(Gln424*)
***FAM161A***	RP	Deletion Exons 13-29	NA	***TUBGCP4***	Chorio-retinopathy	c.1196C>A	p.(Ser399*)
***FRMD7***	Congenital nystagmus	Deletion Entire coding sequence	NA			c.1749G>T(Silent)	p.(Leu582=)
***GPR143***	OCA	Deletion entire coding sequence	NA	***TYR***	OCA	c.221_222del	p.(Val74fs)
***HGSNAT***	RP	Gain Exons 6-18	NA			c.271T>C	p.(Cys91Arg)
***IFT172***	RP	c.3426del	p.(Glu1143fs)	***USH2A***	USH2	c.1417G>A	p.(Trp4725*)
	BBS	c.402+2T>G	NA			c.1551-9T>A	NA
***IFT74***	RP	c.466-2A>G	NA			c.10197C>A	p.(Cys3399*)
***IMPG1***	MD	c.1543_1544dup	p.(Met515llefs*6)			Deletion Exon 69-70	NA
***IMPG2***	RP	Partial Deletion Exons 13-14	NA		RP	c.7454T>A	p.(Leu2485*)
***KCNV2***	RP	c.889_901del	p.(Asp297Serfs*21)			c.8224-1G>A	NA
***KIF11***	Chorio- retinopathy	c.2684dup	p.(Asn895Lysfs*5)			c.8681+2T>C	NA
***MAK***	RP	c.1167del	p.(His389fs)			c.9428A>G	p.(Tyr3143Cys)
		c.1356_1357del	p.(Glu454fs)			c.9441G>A	p.(Trp3147*)
***MERTK***	EOSRD	c.280_81del	p.(Leu94fs)			c.11816_11822dup	p.(Val3942Ilefs*7)
		Deletion Exon 9	NA			c.13018G>A	p.(Gly4340Arg)
***MYO7A***	USH1	c.211_215dup	p.(Leu73Serfs*35)			Deletions Exon 4-72	NA
		c.274del	p.(His92Thrfs*14)			Deletion Exons 20-21	NA
		c.338T>C	p.(Ile113Thr)	***VPS13B***	Cohen syndrome	c.6614T>G	p.Ile2180Arg
		c.3612delC	p.(Ser1205Profs*27)				

*RP* retinitis pigmentosa, *EOSRD* Early onset severe retinal dystrophy, *MD* macular dystrophy, *CHM* choroideremia, *CORD* cone-rod dystrophy, *BBS* Bardet-Biedl syndrome, *FEVR* Familial exudative vitreoretinopathy, *XLRS* X-linked retinoschisis, *CSNB* congenital stationary night blindness, *OA* optic atrophy, *ACHM* achromatopsia, *USH* Usher syndrome.

The variants appeared in similar proportion of individuals with Latino and white self-claimed identity (Supplementary Table 1). The distribution of these previously unreported variants in Argentina is depicted in Fig. 1, where we see that provinces with less mixing between different ethnic populations and European migration (Jujuy, Tucuman and Chaco) have 60%, 43 and 41% of their variants not formerly reported, respectively^4,20^. The transcripts used in this project are detailed on Supplementary Table 3.

### Twenty-one percent diagnostic uplift

One hundred and fifty-six families (156/637) harboring one or more VUS were analyzed in detail, as described in “Methods”.

After such analysis (Supplementary Table 4), 46 families were confirmed as negative, 41 remained in the VUS group, 37 were classified as one (likely) disease-causing variant only, and 32 families (21%) were reclassified to genetically solved (positive).

## Discussion

The disparity in healthcare access between populations and ethnicities is a huge global concern and arguably only increasing^21^. This inequality affects all aspects of medicine, with particular challenges in expensive fields such as advanced therapies and molecular genetics, which are unreachable to huge numbers of people around the world^22,23^. There is a need to advocate for and work towards equal access, not only for ethical purposes but also because more representative global data will increase our understanding of diseases and potentially how different environmental factors play a role.

The study herein is the first large-scale genetic analysis of IED in Argentina and the largest in South America, describing the genetic profile of this understudied population. The diagnostic rate (59%) was in keeping with other countries such as UK^24^, Spain^25^, Poland^26^, Korea^27^, China^28^, and USA^29^. It is noteworthy that next-generation sequencing (NGS)-based panels continue to be a key first-tier test worldwide, with constantly updated panels including complex regions such as *RPGR-*ORF15 and deep intronic areas^24^. These panel tests are currently not covered by most health insurances in Argentina, however, this study further reinforces their relevance as a standard-of-care assessment and their applicability to our region^1^.

The mean age at genetic testing in Argentina was similar to a large cohort in USA (36.5 years versus 37.3)^30^, and younger than other groups in Asia^27,31^. The percentage of individuals with positive family history was similar to other cohorts as well, with consistent no significant age differences between positive and negative family history subcohorts^27,31^. Still, there was an 8-year difference between mean age of onset and diagnosis, and a further 14-year gap until genetic testing. Of course, this represents a significant delay in genetic diagnosis (“the genetic odyssey”), emphasizing the critical need to improve access to affordable genetic testing at the point of clinical diagnosis, rather than several years/decades later.

The most common genes and variants mirrored other large IRD cohorts, with the caveat that our patients were primarily ascertained via a patient group for RP, hence *ABCA4* was the third most common gene instead of the first^25,30–34^. *USH2A* was the most common gene to cause RP, in agreement with other reports;^35,36^ and *RPGR*, the most common gene to cause X-linked RP^37^, as second in prevalence. Interestingly, *RDH12* was the most frequently identified gene causing EOSRD in our cohort, and not *CEP290*, *GUCY2D*, or *CRB1*, as described in Brazil, North Africa, and UK, respectively^16,17,32^. The large variability worldwide regarding EOSRD genes may be due to the small sample size and the potential misclassification of some cases as RP or other rod-cone dystrophies; or maybe a true reflection of genetic diversity globally. *BEST1* appearing as the most frequent gene in MD (with four families), and not *ABCA4* or *PRPH2* (present in three families each), may relate to the selection bias of our sample and Stargardt being a separate clinical category^25,30^.

Our cohort has also provided additional evidence for rare genes with limited cases in the literature, supporting their pathogenicity in IED and their associated phenotypes. *FRMD7* was found in a patient with X-linked congenital nystagmus, as previously reported^38^; biallelic *RTN4IP1* changes were detected in a patient with concomitant RP and optic atrophy, a recently described phenotype^39^; *ARHGEF18* in a patient with autosomal recessive RP^40^; *PRPF6* in a patient with autosomal dominant RP^41^, and *ARSG* in a patient with Usher syndrome type 4^42^. Of note, the variant *ABCA4* p.Asn1868Ile was not reported by the clinical laboratory, hence its linkage with other variants could not be ascertained^43^.

Variant interpretation is key to providing an accurate diagnosis to patients and families, facilitating the best possible clinical management, family counseling/planning, and enabling access to potential gene-based therapies. Particularly in the discipline of rare diseases, every contribution is helpful to better understand the pathophysiology of these conditions. One hundred and fifty-six previously unreported (likely) disease-causing variants were identified, representing 35% of all the variants in the cohort (Table 1). Perhaps unsurprisingly, this is a larger proportion than that reported in well-characterized populations such as those in North America and Europe^44–46^, and closer to values reported in Asian projects^31,47^. A further 38 previously unreported variants were classified as VUS, with more data needed to reclassify them as benign or pathogenic. Similar to proposed disease- and gene-specific guidelines to classify variants^48^, it would be valuable to also introduce population or minority-specific criteria, to be able to recognize population-associated evidence in large-scale genome-based studies.

Certain variants reported herein were not only seen in European alleles (i.e., *USH2A* c.12575 G > A in Spain^49^, *USH2A* c.1751G > T in Italy^50^, *PRPF31* c.371_375del in Germany^51^, *TYR* c.996 G > A in Denmark^52^, *USH2A* c.11864 G > A in UK)^53^, or other American countries (*COL2A1* c.3574 C > T in Brazil^54^, *RPGR* c.1345 C > T in North America^55^), but also in populations from all around the world (*USH2A* c.5329 C > T in Japan^56^, *EYS* c.5450 G > A in a Bedouin tribe in Israel^57^, *FAM161A* c.1003 C > T in Palestine^58^, *CNGB3* c.1148del in Pingelapese islanders of Micronesia^59^, and *USH2A* c.5858 C > G in Tunisia, among others)^60^.

Remarkably, 100% of *CERKL*-associated retinopathy in Argentina was due to c.847 C > T, p(.Arg283*), a variant enriched in European populations, not characterized as prevalent amongst Latinos (https://gnomad.broadinstitute.org/variant/2-182423344-G-A?dataset=gnomad_r2_1)^61^. This is possibly due to European migration to Argentina.

This study’s limitations include its retrospective nature, and that there was a predominant representation of patients with RP compared to other IEDs. Expanding the analysis to include a broader spectrum of disease in the future would benefit patients and scientists alike. Segregation data and detailed clinical information were also limited. There is a restricted testing capacity of NGS-based panels, such as intronic regions remaining untested and the inability to interrogate new genes; tests with larger coverage, such as whole genome sequencing, would be required to uncover a larger proportion of pathogenic variants, although this introduces additional complexities and challenges^62^. Access to testing in this study is likely to have not been uniform across the country and so there may be regions and provinces that are not/underrepresented. Furthermore, patients from rural areas may have traveled to nearby cities to get tested, hence large provinces such as Cordoba and Buenos Aires may include inhabitants from neighboring provinces. There is also limited funding for further required research, such as trio analysis, particularly relevant due to the high incidence of VUS.

In summary, this is the first comprehensive study of the genetic landscape of IRD in Argentina, describing over 150 previously unreported disease-associated variants, and 8 possible founder mutations. *RPGR* and two *USH2A*-exon 13 variants (c.2299del and c.2276 G > T) are frequent in our cohort, in keeping with previous reports^30,63^, and relevant for directed gene therapy clinical trials (NCT04671433, NCT05158296 and NCT05176717). Two unrelated patients with *RPE65*-EOSRD have been treated with Luxturna for the first time in Argentina in 2022, paving the way for more to come. We believe this data improves the understanding of IED genetics in Argentina and will support access to the best possible clinical care for patients, as well as contribute to worldwide registries, and the development of public health policies towards a more equitable access to healthcare.

Moreover, reporting this Argentinian variome for the first time in a cohort this large will contribute to improving the understanding of disease-causing variants, delineating future large-scale population genome projects in South America and, along with other efforts worldwide^64–66^, bring us closer to map human diversity^67,68^.

## Methods

### Medical records review

Medical records of 22 ophthalmology and genetics services throughout 13 provinces in Argentina were reviewed for this retrospective study (Fig. 1). Patients with a clinical diagnosis of an ophthalmic genetic disease and a history of genetic testing were included. The diagnoses were made by trained ophthalmologists and the diagnostic algorithm varied amongst the regions, with a clinical diagnosis based on history and retinal examination in rural areas, and additional multimodal imaging and retinal functional assessments in urban environments. Medical, ophthalmological, and family history was collected.

To reach a diagnostic consensus across centers, RP was defined as a rod-cone dystrophy with onset after 5 years of age; EOSRD, a severe retinal dystrophy presenting before 5 years old;^69^ and Stargardt disease was a category on its own^70^.

This study was performed in accordance with the ethical standards of the Declaration of Helsinki and was approved by the ethics committee of the Argentine Society of Ophthalmology. Written informed consent was obtained in all cases prior to genetic testing. Most of the patients (96%) had genetic testing through a sponsored program by Invitae laboratory (San Francisco, CA, USA), which took place between July 2021 and August 2022. It included an NGS-based IRD panel of 330 genes (https://www.invitae.com/en/providers/test-catalog/test-72100). Twenty patients were tested with an NGS IRD panel of 224 genes (https://mendelics.com.br/en/especialidades/oftalmologia-en/hereditary-retinopathy-panel/), and nine had an older NGS IRD panel of 39 genes (https://dbgen.com/ 2017). Most patients were referred to testing by the RP Argentina Foundation (FARP, www.retinosisargentina.com), hence the sample had a selection bias towards RP.

### Genetic testing analysis

Invitae uses Illumina sequencing technology, with a minimal read depth ≥50x, and aligns the reads to the reference sequence GRCh37. Variants reported as pathogenic and likely pathogenic by the accredited diagnostic laboratory were interpreted as such and not queried. VUS were analyzed by MDV and GA when deemed as possibly disease-causing, based on family history, phenotype, and/or if concurrent with a pathogenic/likely pathogenic change in a candidate recessive gene. This analysis considered the VUS protein effect, familial segregation when available, pathognomonic retinal phenotype when applicable, frequency in the general population (https://gnomad.broadinstitute.org/)^71^, American College of Medical Genetics (ACMG) classification^72^, in silico prediction tools (Revel, MutationTaster, and SpliceAI)^73–76^, conservation score (PhyloP100way)^77^, and their presence in genetic databases (HGMD and ClinVar, Supplementary Table 4). Cases were uplifted to positive when the VUS could be reclassified as likely pathogenic or pathogenic, categorized as negative when no sufficient evidence was found, classified into a “one candidate variant” category if they carried only one pathogenic or likely pathogenic variant in a candidate recessive gene, or placed into a VUS category if the case remained uncertain after analysis. In the exceptional case where the phenotype was pathognomonic of one gene only, and the family history was consistent with the inheritance pattern (Supplementary Table 4, ID 5), PP4 was uplifted to moderate evidence to classify this variant.

GraphPad Prism 8.0.2 (GraphPad Software, San Diego, CA, USA) was implemented for statistical analysis. The threshold of significance was set at *P* < 0.05.

### Reporting summary

Further information on research design is available in the Nature Research Reporting Summary linked to this article.

## Supplementary information

Supplementary Tables 2 & 3

Supplementary Table 1

Supplementary Table 4

Reporting Summary

## Data Availability

The datasets used and/or analyzed during the current study are available from the corresponding author on reasonable request and upon Data Usage Agreement.
