# Supplementary material for: Nationwide genetic analysis of more than 600 families with inherited eye diseases in Argentina
Source: NPJ Genom Med. 2023 May 22;8:8. doi: 10.1038/s41525-023-00352-1 (PMC10202926; doi:10.1038/s41525-023-00352-1)
Supplement: Supplementary file 1 — Supplementary Tables 2 & 3 [file 41525_2023_352_MOESM1_ESM.pdf]

Supplementary Table 2: Previously unreported variants of unknown significance in our cohort.

Gene	Diagnosis	Variant c.	Variant p.
ADGRV1	Usher syndrome	c.17461T>C	p.(Ser5821Pro)
ARHGEF18	RP	c.1276A>C	p.(Ile426Leu)
ARSG	Usher syndrome type 4	c.284G>A	p.(Arg95Gln)
BBS2	RP	c.815G>A	p.(Arg272Gln)
CACNA1F	CSNB	c.3965A>T	p.Glu1376Val
CDHR1	RP	c.862+4A>C	NA
		c.2119G>C	p.(Gly707Arg)
COL11A1	Stickler	c.740C>G	p.(Ala247Gly)
EYS	RP	c.615_616delinsCC	p.(Phe206Leu)
		c.6602T>C	p.(Leu2201Pro)
		c.8959A>G	p.(Ser2987Gly)
		c.8981T>A	p.(Leu2994Gln)
		c.9022A>G	p.(Ser3008Gly)
HK1	RP	c.1550G>A	p.(Arg517Gln)
IFT172	RP	c.3925G>A	p.(Gly1309Ser)
IMPG1	MD	c.2162G>C	p.(Ser721Thr)
IMPG2	RP	c.533G>A	p.(Ser178Asn)
MERTK	RP	c.734C>A	p.(Ser245Tyr)
PDE6B	RP	c.515C>A	p.(Thr172Lys)
		c.1151T>C	p.(Leu384Pro)
PROM1	MD	c.1441G>T	p.(Val481Phe)
	RP	c.1780A>C	p.(Ile594Leu)
PRPF3	RP	c.424-20A>G	NA
PRPF8	RP	c.4103T>C	p.(Leu1368Ser)
RP2	RP	c.535C>T	p.(Pro179Ser)
RPE65	EOSRD	c.998G>A	p.(Gly333Glu)
		c.1444G>T	p.(Asp482Tyr)
RPGR	RP	c.1481G>T	p.(Gly494Val)
USH2A	RP	c.235T>C	p.(Cys79Arg)
		c.436T>C	p.(Ser146Pro)
		c.457T>C	p.(Trp153Arg)
		c.2723T>C	p.(Leu908Ser)
		c.5519G>A	p.(Gly1840Glu)
		c.8371G>C	p.(Val2791Leu)
		c.8846-3C>G	NA
		c.11597C>T	p.(Ala3866Val)
		c.13139C>T	p.(Thr4380Ile)
		c.14257G>A	p.(Val4753Ile)

Abbreviations: RP: retinitis pigmentosa, EOSRD: Early onset severe retinal dystrophy, MD: macular dystrophy, CSNB: congenital stationary night blindness.

Supplementary Table 3: Canonical transcript IDs used for variant nomenclature in this manuscript.

Gene	Transcript
ABCA4	NM_000350.3
ADGRV1	NM_032119.4
AGBL5	NM_021831.6
AHI1	NM_001134831.2
ALMS1	NM_001378454.1
ARHGEF18	NM_001367823.1
ARL6	NM_001278293.3
ARSG	NM_001267727.2
BBS1	NM_024649.5
BBS2	NM_031885.5
BBS4	NM_033028.5
BBS7	NM_176824.3
BEST1	NM_004183.4
CACNA1F	NM_001256789.3
CDH23	NM_022124.6
CDHR1	NM_033100.4
CEP290	NM_025114.4
CERKL	NM_201548.5
CHM	NM_000390.4
CLRN1	NM_174878.3
CNGB1	NM_001297.5
CNGB3	NM_019098.5
COL11A1	NM_001854.4
COL18A1	NM_001379500.1
COL2A1	NM_001844.5
CRB1	NM_201253.3
CRX	NM_000554.6
CWC27	NM_005869.4
CYP4V2	NM_207352.4
EYS	NM_001142800.2
FAM161A	NM_001201543.2
FRMD7	NM_194277.3
FZD4	NM_012193.4
GPR143	NM_000273.3
GUCY2D	NM_000180.4
HGSNAT	NM_152419.3
HK1	NM_000188.3
IFT172	NM_015662.3
IFT74	NM_025103.4
IMPG1	NM_001563.4
IMPG2	NM_016247.4
IQCB1	NM_001023570.4
KCNV2	NM_133497.4

KIF11	NM_004523.4
KIZ	NM_018474.6
LRP5	NM_002335.4
MAK	NM_001242957.3
MERTK	NM_006343.3
MKKS	NM_170784.3
MYO7A	NM_000260.4
NMNAT1	NM_022787.4
NPHP1	NM_001128178.3
NR2E3	NM_014249.4
OCA2	NM_000275.3
OPA1	NM_130837.3
PAX6	NM_001368894.2
PCARE	NM_001029883.3
PDE6A	NM_000440.3
PDE6B	NM_000283.4
PDE6C	NM_006204.4
PHYH	NM_006214.4
PROM1	NM_006017.3
PRPF3	NM_004698.4
PRPF31	NM_015629.4
PRPF6	NM_012469.4
PRPF8	NM_006445.4
PRPH2	NM_000322.5
RDH12	NM_152443.3
REEP6	NM_138393.4
RHO	NM_000539.3
RLBP1	NM_000326.5
RP1	NM_006269.2
RP1L1	NM_178857.6
RP2	NM_006915.3
RPE65	NM_000329.3
RPGR	NM_001034853.2
RPGRIP1	NM_020366.4
RS1	NM_000330.4
RTN41P1	NM_032730.5
SNRNP200	NM_014014.5
TCTN3	NM_015631.6
TSPAN12	NM_012338.4
TTC8	NM_144596.4
TTLL5	NM_015072.5
TUBGCP4	NM_014444.5
TYR	NM_000372.5
USH2A	NM_206933.4
VPS13B	NM_152564.5
WFS1	NM_006005.3
